# Mini- and Micro-Invasive Approaches in Cardiac Surgery: Current Techniques, Outcomes, and Future Perspectives

**DOI:** 10.3390/medicina62010102

**Published:** 2026-01-02

**Authors:** Walter Vignaroli, Barbara Pala, Giuseppe Nasso, Stefano Sechi, Giuseppe Campolongo, Giuseppe Speziale, Emiliano Marco Navarra

**Affiliations:** 1 Department of Cardiac Surgery, San Carlo di Nancy Hospital GVM Care & Research, 00165 Rome, Italy; ssechi@gvmnet.it (S.S.); gcampolongo@gvmnet.it (G.C.); gspeziale@gvmnet.it (G.S.); enavarra@gvmnet.it (E.M.N.); 2 PhD School of Applied Medical-Surgical Sciences, University of Rome Tor Vergata, 00133 Rome, Italy; barbara.pala93@gmail.com; 3 Unit of Cardiology, IDI-IRCCS, Via Monti di Creta, 00167 Rome, Italy; 4 Department of Cardiac Surgery, Anthea Hospital and Santa Maria Hospital GVM Care & Research, 70124 Bari, Italy; gnasso@libero.it; 5 Department of Medicine and Surgery, LUM University, Casamassima, 70124 Bari, Italy; 6 Department of Health and Life Sciences, European University of Rome, 00163 Rome, Italy

**Keywords:** minimally invasive cardiac surgery, mitral repair, aortic valve replacement, hybrid procedure, atrial fibrillation, patient-centered care

## Abstract

Over the past three decades, cardiac surgery has undergone a deep transformation, shifting from full median sternotomy to minimally invasive (MICS) and micro-invasive techniques. These approaches aim to achieve equivalent therapeutic outcomes while reducing surgical trauma, postoperative pain, hospitalization time, and healthcare costs. Minimally invasive strategies are now widely applied to aortic and mitral valve surgery, coronary artery bypass grafting, atrial fibrillation ablation, and combined procedures. Key advancements such as sutureless prostheses, video- and robotic-assisted systems, and enhanced imaging technologies have improved surgical precision and clinical outcomes while promoting faster recovery and superior cosmetic results. Evidence from randomized trials and observational studies demonstrates that MICS provides mortality and morbidity rates comparable to conventional surgery, with additional benefits in high-risk, elderly, and frail patients. Micro-invasive transcatheter interventions, particularly transcatheter aortic valve implantation (TAVI) and transcatheter mitral repair or replacement, have further expanded therapeutic options for patients unsuitable for open-heart surgery. Their success has fostered debate not between conventional and minimally invasive surgery, but between minimally invasive and micro-invasive approaches. Hybrid procedures—combining surgical and percutaneous techniques—exemplify a multidisciplinary evolution aimed at tailoring treatment to patient-specific anatomy, comorbidities, and risk profiles. Despite clear advantages, these techniques present challenges, including a steep learning curve, increased procedural costs, and the requirement for specialized equipment and institutional expertise. Optimal patient selection based on clinical risk assessment and advanced imaging remains essential. Future directions include refinement of robotic platforms, artificial intelligence-based decision support, miniaturization of instruments, and broader validation of emerging technologies in younger and low-risk populations. Minimally and micro-invasive cardiac surgery represent a paradigm shift toward patient-centered care, offering reduced physiological burden, improved functional recovery, and long-term outcomes comparable to conventional techniques. As innovation continues, these approaches are poised to become integral to modern cardiac surgical practice.

## 1. Introduction

Open-heart surgery has historically relied on full median sternotomy to provide broad exposure of the heart and great vessels. Although highly effective and reproducible, this traditional approach is associated with substantial surgical trauma, prolonged postoperative recovery, increased risk of infection, sternal instability or dehiscence, and longer hospital stays. Over the past three decades, the introduction and progressive refinement of minimally invasive cardiac surgery (MICS) and micro-invasive procedures have revolutionized the field. These techniques aim to achieve the same therapeutic goals while significantly reducing physiological stress, postoperative pain, inflammatory response, and overall morbidity [1,2,3,4,5].

In this manuscript, the term MICS refers to surgical procedures performed through limited thoracic access (such as mini-thoracotomy or partial sternotomy), under direct vision and/or endoscopic assistance, while preserving the fundamental principles of conventional cardiac surgery, including cardiopulmonary bypass and surgical implantation of standard prosthetic devices. This approach should be clearly distinguished from micro-invasive procedures (which refer to transcatheter interventions), which are entirely percutaneous, catheter-based procedures performed without surgical exposure or cardiopulmonary bypass and rely on intravascular device delivery rather than surgical implantation.

As surgical expertise and technological resources have evolved, most cardiac procedures have been redefined through minimally invasive access. Today, a wide variety of interventions—including isolated or combined valvular procedures, coronary revascularization, surgical ablation for atrial fibrillation, and left atrial appendage closure—can be safely performed through limited incisions or endoscopic approaches. Consequently, the majority of high-volume cardiac surgery centers are progressively transitioning toward these methods, and only a small number of institutions continue to rely exclusively on conventional sternotomy.

The rationale for adopting minimally invasive strategies extends far beyond patient comfort. Numerous studies have shown that these approaches can reduce hospital costs, shorten intensive care unit stays, lower transfusion requirements, and promote a faster return to daily activities. Additionally, they align with the increasing demand for improved cosmetic outcomes, particularly among younger, active, or professionally exposed patients. Micro-invasive strategies—including transcatheter interventions, hybrid procedures, and image-guided techniques—represent an even more advanced evolutionary step. These approaches facilitate the treatment of complex conditions in fragile or high-risk patients who may not tolerate conventional surgery, thereby expanding therapeutic options.

In contemporary clinical practice, the debate is no longer centered on traditional versus minimally invasive surgery, but rather on the relative roles and advantages of minimally invasive versus micro-invasive procedures. Although the adoption of micro-invasive technologies is rapidly increasing—as highlighted, for example, by the progressive lowering of the age threshold for TAVI indications in the most recent European guidelines [6]—these techniques remain in a dynamic phase of development. Their long-term outcomes, especially in younger patient populations, must be thoroughly validated through robust clinical trials and extended follow-up.

This review aims to provide an in-depth and comprehensive analysis of current mini- and micro-invasive cardiac surgical techniques, emphasizing their indications, clinical applications, perioperative outcomes, limitations, patient selection criteria, and emerging innovations. Through this overview, we aim to clarify the present landscape of minimally invasive cardiac surgery and outline the future directions of this rapidly evolving field.

## 2. Minimally Invasive Cardiac Surgery (MICS): Techniques and Applications 

### 2.1. Aortic Valve and Ascending Aorta Surgery

Minimally invasive aortic valve replacement (MIAVR) is most commonly performed through either a partial upper sternotomy or a right anterior mini-thoracotomy, two approaches that have become the cornerstone of contemporary minimally invasive aortic surgery. The partial upper sternotomy typically requires a 4–6 cm incision in the upper portion of the sternum, often configured as a “J” or inverted “T.” This access provides excellent visualization of the aortic root, ascending aorta, and right atrium while preserving the integrity of the lower sternum. By maintaining greater sternal stability, this approach reduces surgical trauma, postoperative pain, respiratory impairment, and the risk of sternal dehiscence or mediastinal infection. Furthermore, it offers enough working space to allow safe and reproducible cardiopulmonary bypass establishment—most often through central aortic and right atrial cannulation—and ensures adequate myocardial protection through standard cardioplegia delivery techniques. In contrast, right anterior mini-thoracotomy involves lateral thoracic access via the second or third intercostal space, typically at the right sternal border. This method avoids sternal division entirely and is therefore associated with superior chest wall stability, faster mobilization, and improved cosmetic results. It is frequently combined with peripheral arterial and venous cannulation—most commonly through the femoral or axillary vessels—to establish cardiopulmonary bypass in cases where central cannulation is impractical. However, this technique requires advanced surgical expertise, dedicated long-shafted instruments, and, in many cases, videoscopic or endoscopic assistance, which can lengthen the learning curve for new adopters [7,8].

Historically, the first MIAVR procedures were reported in the early 1990s. In 1993, Rao and Kumar performed the first aortic valve replacement via right thoracotomy [9], marking a seminal milestone in cardiac surgery. Shortly thereafter, in 1996, Cosgrove and Sabik introduced the mini-sternotomy approach [10], which rapidly gained acceptance due to its balance between minimally invasive access and procedural reproducibility. These pioneering interventions laid the foundation for the evolution of minimally invasive cardiac surgery and stimulated further technological and procedural advancements.

Over the past two decades, several randomized controlled trials and large observational studies have consistently demonstrated that MIAVR achieves mortality and major morbidity rates comparable to those of conventional full sternotomy. At the same time, MIAVR offers marked reductions in intraoperative blood loss, transfusion requirements, postoperative pain scores, respiratory complications, wound infections, and overall length of hospital stay. These benefits are particularly meaningful in elderly, frail, or comorbid patients, in whom minimizing surgical trauma can significantly accelerate functional recovery, shorten rehabilitation pathways, and improve early postoperative quality of life [7,11,12].

A major innovation contributing to the spread of MIAVR has been the development of sutureless and rapid-deployment prostheses. These next-generation valve systems eliminate the need for multiple annular sutures, thereby substantially reducing aortic cross-clamp and cardiopulmonary bypass times. This efficiency makes MIAVR more accessible in patients with small aortic annuli, severe calcification, or hostile anatomy and has enabled broader implementation of minimally invasive strategies in centers with varying levels of surgical expertise [13,14].

Long-term follow-up data further confirm that prosthetic valve function, structural durability, and patient survival following minimally invasive aortic valve replacement (MIAVR) are comparable to those achieved with conventional AVR performed via full sternotomy, regardless of the type of prosthetic valve implanted. These favorable outcomes should not be interpreted as being exclusively attributable to sutureless or rapid-deployment valves, which, despite facilitating implantation in a minimally invasive setting, have been associated with a higher incidence of paravalvular leak and atrioventricular conduction disturbances requiring permanent pacemaker implantation. Importantly, the development of newer suturing devices and automated knotting systems (such as RAM systems) has significantly simplified the implantation of conventional sutured prosthetic valves through limited access approaches, allowing surgeons to combine the durability and safety profile of standard prostheses with the well-established benefits of MIAVR. As the aging population continues to expand and the global burden of aortic valve disease rises, MIAVR is therefore expected to play an increasingly central role, effectively bridging traditional open surgery and transcatheter therapies while preserving optimal long-term outcomes.

### 2.2. Mitral Valve Surgery

Minimally invasive mitral valve surgery (MIMVS) is most commonly performed through a right mini-thoracotomy, either independently or in combination with video-assisted or robotic technologies. This access route provides a direct trajectory to the mitral valve apparatus and subvalvular structures while eliminating the need for a conventional median sternotomy, thereby reducing musculoskeletal trauma, preserving chest wall integrity, and facilitating a faster postoperative recovery. In video-assisted MIMVS, a thoracoscopic camera is inserted through a dedicated port, offering high-resolution, magnified views of intracardiac anatomy. This improved visualization allows surgeons to perform complex maneuvers with greater precision while maintaining a minimally invasive footprint. In contrast, robotic-assisted techniques rely on articulated instruments that replicate wrist-like movements and high-definition three-dimensional imaging systems, enhancing the surgeon’s dexterity, depth perception, and operative accuracy [15,16,17]. Together, these technological refinements have expanded the boundaries of what can be achieved through small incisions.

Clinical outcomes from multiple high-volume centers consistently demonstrate that MIMVS is associated with reduced intraoperative blood loss, shorter durations of mechanical ventilation, and decreased intensive care unit and overall hospital stay compared with traditional sternotomy-based operations [18]. Patients treated with minimally invasive techniques also tend to experience reduced postoperative pain, earlier mobilization, and faster return to daily activities, contributing to enhanced perioperative quality of life. Although not uniformly observed across all studies, emerging evidence supports a reduction in opioid requirements after MIMVS. In the present study, the significantly lower need for opioid analgesia may reflect reduced surgical trauma, optimized perioperative pathways, or accelerated functional recovery. This finding, while not universal, reinforces the potential of minimally invasive approaches to improve pain control and mitigate opioid-related adverse effects [19].

Importantly, long-term data demonstrate that minimally invasive procedures achieve mitral valve repair rates that match or even surpass those of conventional full sternotomy, particularly in degenerative mitral valve disease, where durable repair is the standard of care [5,20,21]. Robotic-assisted mitral valve surgery, despite requiring substantial training and infrastructure, offers unique advantages in selected patients. The enhanced visualization and precision of the robotic platform facilitate intricate reconstructive interventions such as multi-segment prolapse repair, implantation of artificial chordae, commissural remodeling, and leaflet augmentation. These strengths make robotic-assisted techniques especially valuable in complex anatomical scenarios, where meticulous repair is essential to ensure durable, long-lasting results [22,23,24,25,26,27,28].

In addition to minimally invasive mitral valve procedures, transapical off-pump mitral valve repair has emerged as a feasible alternative for selected high-risk patients. This approach allows direct ventricular access to the mitral valve without the need for cardiopulmonary bypass, facilitating leaflet repair or chordal implantation while minimizing operative trauma. Early clinical experience demonstrates satisfactory procedural success, improvement in mitral regurgitation, and favorable short-term recovery, making it a valuable option for patients unsuitable for conventional surgery or transcatheter edge-to-edge repair [29,30,31,32].

### 2.3. Coronary Artery Bypass Grafting (CABG)

Minimally invasive direct coronary artery bypass (MIDCAB) is performed through a limited left anterior thoracotomy, typically through the fourth or fifth intercostal space, offering a focused yet effective access to the anterior cardiac surface. It is primarily used for single-vessel revascularization, most commonly targeting the left anterior descending (LAD) artery with the left internal mammary artery (LIMA) as the conduit, owing to its excellent long-term patency. By avoiding median sternotomy and cardiopulmonary bypass, MIDCAB reduces surgical trauma, systemic inflammation, and recovery time. The procedure is generally carried out on the beating heart with the aid of mechanical stabilizers, which create a relatively motionless operative field. These characteristics make MIDCAB particularly appealing for patients with isolated LAD disease, high operative risk, or comorbidities such as severe aortic calcification (“porcelain aorta”), chronic obstructive pulmonary disease, frailty, or previous sternotomies [33,34,35,36,37].

Despite its advantages, conventional MIDCAB can be technically demanding due to limited exposure, especially during internal mammary artery harvesting. For this reason, robotic-assisted MIDCAB has emerged as an important evolution of the technique. Robotic systems enable precise and atraumatic harvesting of the internal mammary artery and, in selected centers, can even facilitate the LIMA-to-LAD anastomosis. Three-dimensional magnified visualization, tremor filtration, and articulated instruments enhance surgical dexterity and reduce chest wall trauma. Clinically, this translates into lower postoperative pain, fewer wound-related complications, improved respiratory mechanics, and superior cosmetic outcomes, while maintaining graft patency and survival rates comparable to those of traditional MIDCAB. However, robotic-assisted MIDCAB is associated with longer operative times during the learning curve, increased procedural costs, and the need for specialized expertise and institutional resources, making it most suitable for high-volume centers [38,39,40,41,42,43].

Totally endoscopic coronary artery bypass (TECAB) represents a further technological advancement, enabling complete coronary revascularization through small thoracic ports without requiring thoracotomy or sternotomy. TECAB can be performed either on-pump with cardioplegic arrest or off-pump on a beating heart, depending on anatomical considerations and surgeon preference. In the study by Nisivaco et al., total endoscopic hybrid coronary revascularization was demonstrated to be a safe and effective strategy, associated with low perioperative morbidity and mortality, rapid postoperative recovery, and favorable mid- to long-term clinical outcomes. High graft patency rates, sustained freedom from angina, and a low need for repeat revascularization were reported, supporting the role of this minimally invasive approach in carefully selected patients. Robotic platforms offer enhanced visualization, tremor reduction, and dexterous micro-instruments, allowing multi-vessel grafting in carefully selected patients and potentially expanding its indications in the future [44,45].

Clinical outcomes for both MIDCAB and TECAB consistently demonstrate several perioperative advantages compared to conventional coronary artery bypass grafting (CABG), including reduced postoperative pain, lower rates of wound infection and mediastinitis, decreased transfusion requirements, shorter intensive care unit and hospital stays, and faster return to daily activities. Patients undergoing minimally invasive procedures also report better cosmetic satisfaction and improved postoperative quality of life. Importantly, meta-analyses and long-term follow-up studies confirm that LIMA-to-LAD graft patency and long-term survival are comparable to those achieved with conventional CABG, supporting the role of these minimally invasive strategies as effective and durable alternatives in appropriately selected patients [46].

Even traditional coronary surgery has sought to become less invasive through techniques such as endoscopic vein harvest (EVH) and smaller incisions, bridging the gap between conventional and minimally invasive approaches. EVH is today the standard of care, offering comparable graft patency to open techniques while minimizing wound complications, postoperative pain, and hospital stay [47,48,49].

### 2.4. Atrial Fibrillation

Surgical ablation, on the other hand, allows for more extensive and anatomically precise lesion sets, with direct visualization and transmural energy delivery. Yet, due to its invasive nature and perceived risk profile, it has not been widely adopted as a first-line treatment.

MICS has significantly evolved over the past few decades, offering alternative approaches for the treatment of atrial fibrillation (AF) as well, through surgical ablation techniques. These procedures, which include thoracoscopic and mini-thoracotomy, aim to replicate the biatrial Cox–Maze lesion set while minimizing surgical trauma [50,51].

Advances in energy sources—such as radiofrequency, cryoablation, and high-intensity focused ultrasound—have improved transmurality, procedural safety, and long-term rhythm outcomes. Compared to conventional median sternotomy, minimally invasive ablation is associated with reduced postoperative pain, shorter hospital stays, lower transfusion rates, and enhanced cosmetic results, without compromising procedural efficacy.

Despite these advantages, challenges remain in terms of patient selection, procedural complexity, and the need for standardized lesion sets and outcome reporting. Continued advancements in imaging, device technology, and multidisciplinary collaboration are expected to further refine the role of minimally invasive surgical ablation in the contemporary management of atrial fibrillation (Table 1 and Table 2).

## 3. Micro-Invasive Procedures

Transcatheter interventions have radically reshaped the landscape of cardiac surgery, offering less invasive therapeutic alternatives for patients considered high-risk or unsuitable for conventional open-heart procedures. Among these innovations, transcatheter aortic valve implantation (TAVI) stands out as one of the most transformative developments of the past two decades. Initially reserved for inoperable patients with severe aortic stenosis, TAVI has progressively evolved into the preferred treatment option for elderly individuals and those with intermediate surgical risk. Randomized controlled trials such as PARTNER 1, PARTNER 2, and SURTAVI have demonstrated that TAVI achieves survival outcomes and valve hemodynamics comparable to, and in selected populations even superior to, those obtained with surgical aortic valve replacement (SAVR) [38,39,40]. Furthermore, subsequent studies—including PARTNER 3 and the Evolut Low-Risk Trial—have provided robust evidence that TAVI is non-inferior in appropriately selected low-risk patients [52,53,54]. Long-term data from PARTNER 3 at 5 years show similar composite outcomes of death, stroke, and rehospitalization between TAVI and SAVR, with stable hemodynamic performance and low rates of valve failure, thereby supporting the continued expansion of TAVI indications and reinforcing its role as a mainstream therapeutic modality [55] In parallel, transcatheter mitral valve interventions have significantly broadened the treatment landscape for mitral regurgitation. Among these, transcatheter edge-to-edge repair (TEER), best exemplified by the MitraClip system, has become the most widely adopted TMVR strategy. By reproducing the surgical Alfieri stitch, the device approximates the anterior and posterior leaflets, effectively reducing regurgitation while preserving the native valve apparatus. The landmark COAPT trial demonstrated substantial reductions in heart failure hospitalizations and all-cause mortality in patients with secondary mitral regurgitation treated with MitraClip in addition to guideline-directed medical therapy compared with medical therapy alone [56]. Beyond TEER, emerging technologies—including transcatheter mitral valve replacement (TMVR) systems such as Tendyne and Intrepid, as well as transcatheter chordal repair devices—are under active clinical investigation. Early feasibility studies have shown promising procedural success and symptomatic improvement, suggesting that these modalities may soon complement or expand current therapeutic strategies [57,58].

Transcatheter therapies for severe tricuspid regurgitation have rapidly evolved, transforming the management of a condition historically labeled the “forgotten valve” due to limited surgical options and high operative risk. The first FDA-approved transcatheter tricuspid valve replacement (TTVR) system, EVOQUE (Edwards Lifesciences) [59], has demonstrated compelling safety and effectiveness data, with the pivotal TRISCEND II trial showing high rates of successful implantation and near-complete elimination of regurgitation at one year, significant improvements in symptoms, functional status, and quality of life compared with optimal medical therapy alone, and favorable trends in mortality and heart failure hospitalizations [60,61]. In addition to TTVR, FDA-approved edge-to-edge repair systems such as TriClip and PASCAL have shown clinical benefits in reducing regurgitation and improving outcomes in high-risk patients with symptomatic severe tricuspid disease. Collectively, these percutaneous approaches offer viable alternatives to surgery in appropriately selected patients, expanding the therapeutic armamentarium for tricuspid valve disease and improving patient-centered outcomes [62,63].

Overall, the rapid and continuous expansion of transcatheter therapies reflects a profound paradigm shift in contemporary cardiovascular care. This evolution has been driven by advances in device engineering, enhanced multimodality imaging, improved patient selection criteria, and an increasing emphasis on minimally invasive, patient-centered treatment strategies. As technologies continue to mature and long-term data accumulate, transcatheter interventions are poised to play an increasingly central role in the management of structural heart disease across a wide spectrum of risk profiles.

## 4. Hybrid Techniques

Hybrid techniques represent a new tool in the hands of medicine, allowing the treatment of increasingly high-risk patients who cannot benefit from a single procedure to achieve a comprehensive therapy. Their use can be extended to valvular and coronary procedures, as well as to the treatment of atrial fibrillation.

Hybrid coronary revascularization has emerged as an effective complementary strategy, combining the durability of surgical LIMA-to-LAD grafting with the minimally invasive nature and versatility of percutaneous coronary intervention (PCI) for non-LAD lesions. This hybrid revascularization strategy, combining LIMA–LAD bypass with PCI to non-LAD vessels, is particularly advantageous in polymorbid patients with left main coronary artery disease, as it couples the proven long-term patency and survival benefit of the LIMA–LAD graft with the reduced invasiveness and procedural flexibility of percutaneous coronary intervention for complete revascularization. It may be performed simultaneously in a hybrid operating room or as a staged procedure. Early data indicate that hybrid revascularization offers reduced perioperative morbidity, shorter recovery time, and comparable long-term outcomes to conventional multivessel CABG, while preserving the survival benefits associated with arterial grafting [42].

Furthermore, hybrid strategies have emerged as a promising option for patients with persistent or long-standing persistent AF, particularly after failed catheter ablation. Recent trials, including CONVERGE and CEASE-AF, have demonstrated superior arrhythmia-free survival and fewer repeat procedures compared with catheter ablation alone, supporting the benefit of complete transmural lesion sets achieved via combined epicardial and endocardial approaches [64,65,66,67,68,69,70,71,72].

The emergence of hybrid procedures—combining surgical and transcatheter techniques—exemplifies the evolution toward a multidisciplinary heart team approach. This model emphasizes close collaboration among cardiac surgeons, interventional cardiologists, anesthesiologists, and imaging specialists. Hybrid interventions facilitate personalized treatment strategies tailored to anatomical complexity, patient frailty, comorbidities, and previous cardiac surgeries. These approaches are also particularly beneficial in patients with complex valve pathology, heavily calcified aortas, prior sternotomies, or severe comorbid conditions where isolated surgical or transcatheter methods alone may be inadequate or carry excessive risk [73].

## 5. Discussion

### 5.1. Clinical Outcomes and Evidence

A substantial body of literature—including randomized controlled trials, propensity-matched analyses, and large meta-analyses—demonstrates that minimally invasive cardiac surgery (MICS) is both safe and effective across a wide range of procedures. Operative mortality rates are consistently shown to be comparable to those of conventional full sternotomy, even when minimally invasive approaches are applied in high-risk populations. Studies report several perioperative advantages, including reduced intraoperative blood loss, lower transfusion requirements, shorter cardiopulmonary bypass and cross-clamp times in experienced centers, and decreased duration of mechanical ventilation. These improvements translate into shorter intensive care unit (ICU) and overall hospital stays, faster postoperative mobilization, and earlier return to functional independence.

Patients undergoing minimally invasive interventions also report superior quality-of-life outcomes. Reduced postoperative pain, diminished impairment of chest wall mechanics, and better cosmetic satisfaction—all direct or indirect consequences of smaller incisions—contribute to heightened postoperative comfort. Several patient-reported outcome measures (PROMs) highlight increased satisfaction with body image, improved early physical function, and faster return to normal daily activities.

Despite these advantages, important limitations remain. Minimally invasive procedures may be associated with longer operative times or extended cardiopulmonary bypass duration during the learning curve. The restricted operative field and reduced tactile feedback can pose technical challenges, especially in complex cases requiring extensive reconstruction. Conversion to full sternotomy—while infrequent—remains a possibility in cases of inadequate exposure, unexpected anatomical findings, or intraoperative complications. Therefore, proper patient selection and surgical expertise remain essential for maximizing clinical benefits and minimizing risks.

### 5.2. Patient Selection

Appropriate patient selection is a cornerstone of success in both mini- and micro-invasive cardiac surgery. Preoperative assessment should begin with a comprehensive clinical history and physical examination focused on identifying factors that may influence procedural feasibility, such as obesity, frailty, prior thoracic surgery, or chest wall abnormalities. Detailed imaging assessment is fundamental. Computed tomography (CT) angiography is crucial for evaluating vascular access, assessing aortic anatomy, and identifying potential calcifications that could complicate minimally invasive entry points. Transthoracic and transesophageal echocardiography provide essential information about valve morphology, ventricular function, and suitability for repair versus replacement.

Pulmonary function testing helps identify patients whose ventilatory reserve may be compromised by thoracotomy or single-lung ventilation, while coronary CT angiography or invasive coronary angiography assists in determining the need for concomitant coronary revascularization. Risk stratification tools such as the EuroSCORE II and the STS score assist in estimating surgical risk and guiding the decision between traditional, minimally invasive, and transcatheter approaches. Elderly or frail patients, as well as those with significant comorbidities—including porcelain aorta, chronic lung disease, or chronic kidney disease—frequently derive substantial benefit from micro-invasive transcatheter interventions [74,75,76].

### 5.3. Future Perspectives

The future evolution of minimally invasive cardiac surgery (MICS) will be shaped by rapid advancements in technology, imaging, and procedural methodology, ultimately broadening the scope and safety of these interventions. Next-generation robotic platforms are expected to offer even greater instrument articulation, superior ergonomics, and enhanced haptic feedback, enabling surgeons to perform increasingly complex valve repairs, coronary anastomoses, and intracardiac procedures through very small thoracic ports. These systems will likely support more intuitive and precise movements, reducing operative time and improving reproducibility while minimizing patient trauma.

The integration of advanced intraoperative imaging modalities—including real-time three-dimensional transesophageal echocardiography (3D TEE), augmented reality overlays, image-fusion systems, and high-definition fluoroscopy—will allow unprecedented anatomical precision. Surgeons will be able to navigate complex cardiac structures with confidence, optimize procedural planning, and dynamically adjust strategies during interventions. The combination of imaging with robotic guidance may also reduce the learning curve associated with technically demanding procedures and improve outcomes in centers adopting MICS.

Artificial intelligence (AI) and machine learning (ML) are poised to revolutionize multiple facets of minimally invasive cardiac care. Predictive algorithms may enhance patient selection by accurately identifying candidates best suited for minimally invasive versus transcatheter therapies, estimating individualized procedural risks, and anticipating potential complications. During surgery, AI-assisted platforms could provide real-time decision support, optimize instrument positioning, and suggest corrective maneuvers based on intraoperative feedback, thereby enhancing safety and efficacy [77,78,79].

Moreover, ongoing miniaturization of surgical instruments and the development of innovative prosthetic devices—including sutureless and rapid-deployment valves, as well as next-generation transcatheter systems—will expand the range of pathologies amenable to micro-invasive approaches. These technological innovations are likely to improve procedural durability, reduce operative times, and shorten recovery, while facilitating interventions in high-risk, elderly, or comorbid patients.

Collectively, these advances signal a shift toward a more precision-based, patient-tailored paradigm in cardiac surgery, where minimally invasive strategies can rival traditional open approaches in safety and efficacy while offering substantial benefits in terms of recovery, quality of life, and long-term outcomes. As technology, imaging, and data-driven decision-making continue to evolve, the boundaries of what can be achieved through minimally invasive cardiac interventions will expand, potentially redefining the standard of care over the next decade.

### 5.4. Telesurgery and Emerging Robotic Systems

Advances in telesurgery and next-generation robotic platforms are poised to further transform minimally invasive cardiac surgery. Modern systems offer enhanced instrument articulation, three-dimensional visualization, and improved haptic feedback, enabling surgeons to perform complex repairs through smaller incisions with greater precision. Telesurgical technologies also allow remote proctoring and potentially long-distance surgical interventions, expanding access to specialized care and facilitating real-time collaboration between expert centers. Early clinical experiences suggest that these innovations can improve procedural efficiency, reduce operative trauma, and enhance patient outcomes, particularly in high-risk or anatomically complex cases [80,81,82].

### 5.5. Clinical Implications and Future Directions

In the contemporary management of structural and coronary heart disease, the clinical relevance of minimally invasive and micro-invasive cardiac interventions extends well beyond the demonstration of technical feasibility or short-term perioperative advantages. Following the expansion of indications for transcatheter therapies after the PARTNER 3 and Evolut Low Risk trials, the central challenge has shifted toward appropriate patient selection, particularly among elderly, frail, and multimorbid populations [52,83]. Chronological age alone is no longer sufficient to guide therapeutic decisions; instead, frailty assessment, anatomical complexity, functional status, comorbidity burden, life expectancy, and patient preferences must be systematically integrated into contemporary treatment algorithms [84,85]. Within this framework, minimally invasive surgery, transcatheter interventions, and hybrid strategies should be interpreted as complementary modalities along a continuum of care rather than as competing or mutually exclusive techniques.

In addition to clinical outcomes, economic evaluations increasingly support the value of TAVI in low- and intermediate-risk patients, showing that despite higher upfront procedural costs, TAVI is generally cost-effective compared with SAVR by yielding meaningful gains in quality-adjusted life years (QALYs) and acceptable incremental cost-effectiveness ratios in these populations, and in some analyses even dominant in intermediate-risk patients [86,87].

A major clinical implication of this evolution is the growing centrality of multidisciplinary Heart Team-based decision-making. Current international guidelines emphasize that individualized treatment selection should balance procedural minimalism and early recovery against long-term considerations such as prosthetic durability, access-related complications, and the feasibility of future reinterventions, particularly in patients with long anticipated survival [85,88]. These issues are especially relevant in low- and intermediate-risk cohorts, where enthusiasm for transcatheter solutions must be tempered by unresolved questions regarding valve longevity and lifetime management strategies. Similarly, while minimally invasive and robotic surgical techniques have reached technical maturity, their clinical benefit remains highly dependent on institutional volume, operator expertise, and structured training pathways, reinforcing the need for selective rather than indiscriminate adoption.

From a broader health systems perspective, cost considerations and resource allocation further influence the real-world implementation of minimally invasive and micro-invasive strategies. Although these approaches may reduce hospital length of stay and early morbidity, their upfront costs, infrastructural demands, and learning curves vary substantially across healthcare systems. Hybrid procedures, combining surgical and percutaneous techniques either concomitantly or in a staged fashion, may provide tailored solutions for selected high-risk or complex patients, but they require advanced organizational models, close multidisciplinary coordination, and integrated imaging and procedural planning. Consequently, their long-term sustainability depends not only on technological innovation but also on institutional capacity and system-level optimization.

Looking ahead, future progress in the field is likely to be driven less by incremental procedural novelty and more by refined clinical algorithms that incorporate frailty assessment, anatomical stratification, and patient-reported outcomes into standardized yet flexible care pathways. Longer-term follow-up from randomized trials, large-scale registries, and comparative effectiveness studies focused on real-world populations will be essential to address ongoing controversies related to durability, quality of life, and cost-effectiveness. In this evolving landscape, interpretative narrative syntheses that prioritize clinical reasoning, multidisciplinary integration, and patient-centered strategy selection may offer practical value by supporting Heart Team discussions and aligning therapeutic choices with individualized patient goals.

### 5.6. Limitations and Challenges

Despite their promise, these techniques present unique challenges. Mastery of minimally invasive procedures requires a steep learning curve, often necessitating specialized training programs, high procedural volumes, and mentorship in experienced centers. Not all patients are suitable candidates; severe chest wall deformities, intrathoracic adhesions from prior surgery, extensive calcification of the aorta or annulus, and advanced pulmonary disease can significantly limit feasibility. Institutional support—including access to robotic platforms, hybrid operating rooms, and a multidisciplinary heart team—is essential for optimal outcomes. Finally, the economic implications of implementing MICS and micro-invasive programs, including equipment costs and required infrastructure, must be carefully considered.

## 6. Conclusions

Mini- and micro-invasive approaches represent a major paradigm shift in contemporary cardiac surgery, offering the potential to reduce surgical trauma while maintaining or even improving clinical outcomes. Evidence robustly supports their safety, efficacy, and patient-centered benefits in both valve and coronary procedures. With ongoing technological innovation, expanding surgeon expertise, and deepening integration with advanced imaging and transcatheter therapies, these approaches are poised to become increasingly central to cardiac surgical practice. Ultimately, minimally invasive and micro-invasive strategies promise to deliver improved recovery, enhanced quality of life, and durable long-term results for a growing and diverse patient population.

## Figures and Tables

**Table 1:** Advantages and disadvantages of minimally invasive and micro-invasive cardiac techniques.

Characteristic	Minimally Invasive Cardiac Surgery (MICS)	Micro-Invasive/Transcatheter Techniques
Main Advantages	• Reduced surgical trauma and blood loss • Less postoperative pain • Faster mobilization and recovery • Lower infection and wound complication rates • Superior cosmetic results • Suitable for complex valvular and coronary procedures	• No sternotomy or major thoracic incision • No cardiopulmonary bypass in most cases • Ultra-rapid recovery and shorter hospitalization • Ideal for elderly, frail, or high-risk patients • Lower overall physiological burden
Limitations/Disadvantages	• Steep learning curve • Limited operative exposure • Possible longer operative and CPB times during early experience • Need for dedicated equipment and trained teams • Risk of conversion to sternotomy	• Durability still under evaluation, especially in younger patients • Anatomical constraints (vascular access, calcification, annular size) • High device and procedural costs • Requires advanced imaging and multidisciplinary Heart Team expertise
Ideal Candidates	Low- to intermediate-risk patients; younger individuals; candidates for complex reparative procedures	Elderly, frail, or high-risk surgical patients; patients with significant comorbidities
Clinical Outcomes	Comparable mortality and morbidity to full sternotomy, with superior postoperative recovery	Non-inferior or superior outcomes compared to surgery in selected populations (e.g., TAVI, TEER)
Technical Requirements	High-volume centers; experienced minimally invasive surgical teams	Hybrid operating room; advanced imaging integration; multidisciplinary expertise

Legend: MICS = minimally invasive cardiac surgery; CPB = cardiopulmonary bypass; TAVI = transcatheter aortic valve implantation; TEER = transcatheter edge-to-edge repair.

**Table 2:** Overview of mini-invasive and micro-invasive approaches in contemporary cardiac surgery.

Category	Approach	Description and Main Indications
Aortic Valve—MICS	Partial Upper Mini-sternotomy	Limited J- or inverted-T sternotomy with reliable exposure of the aortic root; indicated for aortic valve replacement.
	Right Anterior Mini-thoracotomy	Avoids sternal division; preferred in patients with high aortic position or when improved cosmetic results are desired.
Mitral Valve—MICS	Right Mini-thoracotomy	Standard access for minimally invasive mitral repair and replacement; suitable for degenerative disease and selected complex cases.
	Video-Assisted MIMVS	Offers magnified visualization of leaflet and subvalvular structures; enhances precision during repair.
	Robotic-Assisted Mitral Surgery	Provides 3D visualization and articulated instruments; ideal for complex multi-segment repair.
Coronary Surgery—MICS	MIDCAB	Off-pump LIMA-to-LAD grafting via small left thoracotomy; indicated for isolated LAD disease.
	Robotic MIDCAB	Robotic harvesting of the LIMA and, in selected cases, robotic-assisted anastomosis; reduces chest wall trauma.
	TECAB	Fully endoscopic coronary bypass via ports without thoracotomy; suitable for selected multivessel disease.
Atrial Fibrillation—MICS	Bilateral Thoracoscopic Ablation	Replicates bi-atrial Cox-Maze IV lesion set using RF or cryo-energy; indicated for persistent or long-standing AF.
	Right Mini-thoracotomy Maze	Single-sided access with simplified lesion set; used in selected AF patients.
Micro-Invasive (Transcatheter)	TAVI (TF, TAx, TA)	Transfemoral preferred; indicated for severe aortic stenosis in high-, intermediate-, and selected low-risk patients.
	TEER (MitraClip, PASCAL)	Edge-to-edge leaflet approximation; indicated for secondary MR and selected primary MR.
	TMVR (Tendyne, Intrepid)	Transcatheter mitral valve replacement for anatomically unsuitable repair cases.
	Transcatheter Tricuspid Therapies	Includes TriClip, PASCAL, Cardioband, and orthotopic TVR; indicated for severe symptomatic TR in high-risk patients.
Hybrid Procedures	Hybrid Coronary Revascularization	Combines LIMA-LAD surgical graft with PCI on non-LAD vessels; useful in multivessel disease with high surgical risk.
	Hybrid AF Ablation	Combines thoracoscopic ablation with catheter mapping and endocardial completion; superior to stand-alone procedures in selected cases.

Legend: LIMA = left internal mammary artery; LAD = left anterior descending artery; MIDCAB = minimally invasive direct coronary artery bypass; TECAB = totally endoscopic coronary artery bypass; AF = atrial fibrillation; RF = radiofrequency; TF = transfemoral; TAx = trans-axillary; TA = trans-apical; MR = mitral regurgitation; TR = tricuspid regurgitation.

## Data Availability

No new data were created or analyzed in this study.

## References

[1] Miceli A., Murzi M., Gilmanov D., Fugà R., Ferrarini M., Solinas M., Glauber M. (2014). Minimally invasive aortic valve replacement using right minithoracotomy is associated with better outcomes than ministernotomy. J. Thorac. Cardiovasc. Surg..

[2] Akowuah E.F., Maier R.H., Hancock H.C., Kharatikoopaei E., Vale L., Fernandez-Garcia C., Ogundimu E., Wagnild J., Mathias A., Walmsley Z. (2023). Minithoracotomy vs Conventional Sternotomy for Mitral Valve Repair: A Randomized Clinical Trial. JAMA.

[3] Acker M.A., Parides M.K., Perrault L.P., Moskowitz A.J., Gelijns A.C., Voisine P., Smith P.K., Hung J.W., Blackstone E.H., Puskas J.D. (2014). Mitral-Valve Repair versus Replacement for Severe Ischemic Mitral Regurgitation. N. Engl. J. Med..

[4] Magruder J.T., Holst K.A., Thourani V.H. (2023). Small, Smaller, Smallest: Minimally Invasive Approaches to Aortic Valve Disease. Ann. Thorac. Surg..

[5] Axtell A.L., Moonsamy P., Dal-Bianco J.P., Passeri J.J., Sundt T.M., Melnitchouk S. (2020). Minimally Invasive Nonresectional Mitral Valve Repair Can Be Performed With Excellent Outcomes. Ann. Thorac. Surg..

[6] Praz F., Borger M.A., Lanz J., Marin-Cuartas M., Abreu A., Adamo M., Marsan N.A., Barili F., Bonaros N., Cosyns B. (2025). 2025 ESC/EACTS Guidelines for the management of valvular heart disease. Eur. J. Cardiothorac. Surg..

[7] Awad A.K., Ahmed A., Mathew D.M., Varghese K.S., Mathew S.M., Khaja S., Newell P.C., Okoh A.K., Hirji S. (2024). Minimally invasive, surgical, and transcatheter aortic valve replacement: A network meta-analysis. J. Cardiol..

[8] Abdelaal S.A., Abdelrahim N.A., Mamdouh M., Ahmed N., Ahmed T.R., Hefnawy M.T., Alaqori L.K., Abozaid M. (2023). Comparative effects of minimally invasive approaches vs. conventional for obese patients undergoing aortic valve replacement: A systematic review and network meta-analysis. BMC Cardiovasc. Disord..

[9] Rao P.N., Kumar A.S. (1993). Brief Communication Aortic Valve Replacement through Right Thoracotomy. Texas Heart Inst. J..

[10] Cosgrove D.M., Sabik J.F. (1996). Minimally invasive approach for aortic valve operations. Ann. Thorac. Surg..

[11] Jahangiri M., Hussain A., Akowuah E. (2019). Minimally invasive surgical aortic valve replacement. Heart.

[12] El-Andari R., Fialka N.M., Shan S., White A., Manikala V.K., Wang S. (2024). Aortic Valve Replacement: Is Minimally Invasive Really Better? A Contemporary Systematic Review and Meta-Analysis. Cardiol. Rev..

[13] Pollari F., Fischlein T. (2020). Minimally invasive sutureless and rapid deployment aortic valve replacement: The new benchmark for aortic valve surgery?. Ann. Cardiothorac. Surg..

[14] Di Bacco L., Miceli A., Glauber M. (2021). Minimally invasive aortic valve surgery. J. Thorac. Dis..

[15] Paparella D., Fattouch K., Moscarelli M., Santarpino G., Nasso G., Guida P., Margari V., Martinelli L., Coppola R., Albertini A. (2020). Current trends in mitral valve surgery: A multicenter national comparison between full-sternotomy and minimally-invasive approach. Int. J. Cardiol..

[16] Speziale G., Moscarelli M., Di Bari N., Bonifazi R., Salardino M., De Donatis T., Nasso G. (2018). A Simplified Technique for Complex Mitral Valve Regurgitation by a Minimally Invasive Approach. Ann. Thorac. Surg..

[17] Moscarelli M., Fattouch K., Gaudino M., Nasso G., Paparella D., Punjabi P., Athanasiou T., Benedetto U., Angelini G.D., Santarpino G. (2020). Minimal Access Versus Sternotomy for Complex Mitral Valve Repair: A Meta-Analysis. Ann. Thorac. Surg..

[18] Moscarelli M., Fattouch K., Casula R., Speziale G., Lancellotti P., Athanasiou T. (2016). What is the role of minimally invasive mitral valve surgery in high-risk patients? a meta-analysis of observational studies. Ann. Thorac. Surg..

[19] Jahanian S., Arghami A., Wittwer E.D., King K.S., Daly R.C., Dearani J.A., Rowse P.G., Crestanello J.A., Schaff H.V. (2023). Does Minimally Invasive Mitral Valve Repair Mean Less Postoperative Pain?. Ann. Thorac. Surg..

[20] Feirer N., Kornyeva A., Lang M., Sideris K., Voss B., Krane M., Lange R., Vitanova K. (2022). Non-robotic minimally invasive mitral valve repair: A 20-year single-centre experience. Eur. J. Cardiothorac. Surg..

[21] Sündermann S.H., Czerny M., Falk V. (2015). Open vs. Minimally Invasive Mitral Valve Surgery: Surgical Technique, Indications and Results. Cardiovasc. Eng. Technol..

[22] Navarra E., Mastrobuoni S., De Kerchove L., Glineur D., Watremez C., Van Dyck M., El Khoury G., Noirhomme P. (2017). Robotic mitral valve repair: A European single-centre experience. Interact. Cardiovasc. Thorac. Surg..

[23] Williams M.L., Eranki A., Mamo A., Wilson-Smith A., Hwang B., Sugunesegran R., Yan T., Navarra E., Guy T.S., Bonatti J. (2022). Systematic review and meta-analysis of mid-term survival, reoperation, and recurrent mitral regurgitation for robotic-assisted mitral valve repair. Ann. Cardiothorac. Surg..

[24] Klepper M., Noirhomme P., de Kerchove L., Mastrobuoni S., Spadaccio C., Lemaire G., El Khoury G., Navarra E. (2022). Robotic mitral valve repair: A single center experience over a 7-year period. J. Card. Surg..

[25] Lehr E.J., Sloane Guy T., Smith R.L., Grossi E.A., Shemin R.J., Rodriguez E., Ailawadi G., Agnihotri A.K., Fayers T.M., Clark Hargrove W. (2016). Minimally Invasive Mitral Valve Surgery III: Training and Robotic-Assisted Approaches. Innovations.

[26] Marin Cuartas M., Javadikasgari H., Pfannmueller B., Seeburger J., Gillinov A.M., Suri R.M., Borger M.A. (2017). Mitral valve repair: Robotic and other minimally invasive approaches. Prog. Cardiovasc. Dis..

[27] Bates M.J., Chitwood W.R. (2021). Minimally invasive and robotic approaches to mitral valve surgery: Transthoracic aortic crossclamping is optimal. JTCVS Tech..

[28] Van Praet K.M., Kempfert J., Jacobs S., Stamm C., Akansel S., Kofler M., Sündermann S.H., Nazari Shafti T.Z., Jakobs K., Holzendorf S. (2021). Mitral valve surgery: Current status and future prospects of the minimally invasive approach. Expert. Rev. Med. Devices.

[29] Colli A., Manzan E., Zucchetta F., Bizzotto E., Besola L., Bagozzi L., Bellu R., Sarais C., Pittarello D., Gerosa G. (2016). Transapical off-pump mitral valve repair with Neochord implantation: Early clinical results. Int. J. Cardiol..

[30] Ahmed A., Abdel-Aziz T.A., AlAsaad M.M.R., Majthoob M. (2021). Transapical off-pump mitral valve repair with NeoChord implantation: A systematic review. J. Card. Surg..

[31] Gerosa G., Nadali M., Longinotti L., Ponzoni M., Caraffa R., Fiocco A., Pradegan N., Besola L., Onofrio A.D., Bizzotto E. (2021). Transapical off-pump echo-guided mitral valve repair with neochordae implantation mid-term outcomes. Ann. Cardiothorac. Surg..

[32] D’Onofrio A., Fiocco A., Nadali M., Mastro F., Aruta P., Lorenzoni G., Pittarello D., Gerosa G., Evangelista G., Longinotti L. (2023). Outcomes of transapical mitral valve repair with neochordae implantation. J. Thorac. Cardiovasc. Surg..

[33] Xu Y., Li Y., Bao W., Qiu S. (2020). MIDCAB versus off-pump CABG: Comparative study. Hell. J. Cardiol..

[34] Manuel L., Fong L.S., Betts K., Bassin L., Wolfenden H. (2022). LIMA to LAD grafting returns patient survival to age-matched population: 20-year outcomes of MIDCAB surgery. Interact. Cardiovasc. Thorac. Surg..

[35] Verevkin A., Von Aspern K., Tolboom H., Gadelkarim I., Etz C., Misfeld M., Borger M.A., Davierwala P.M. (2024). Total Arterial Multivessel Minimally Invasive Coronary Artery Bypass Surgery: 5-Year Outcomes. Ann. Thorac. Surg..

[36] Gong Y., Ding T., Wang X., Cui Z., Zhao H., Wu S., Fu Y., Yang H., Ling Y. (2025). Minimally Invasive vs Conventional Coronary Bypass Surgery for Multivessel Coronary Disease. Ann. Thorac. Surg. Short. Rep..

[37] Zhang L., Gao Q., Zhang S., Liu G., Lian B., Chen Y., Shi Y. (2025). Clinical outcomes of multivessel disease patients after off-pump minimally invasive coronary surgery. J. Cardiothorac. Surg..

[38] Gofus J., Cerny S., Shahin Y., Sorm Z., Vobornik M., Smolak P., Sethi A., Marcinov S., Karalko M., Chek J. (2022). Robot-assisted vs. conventional MIDCAB: A propensity-matched analysis. Front. Cardiovasc. Med..

[39] Pettinari M., Navarra E., Noirhomme P., Gutermann H. (2017). The state of robotic cardiac surgery in Europe. Ann. Cardiothorac. Surg..

[40] Pettinari M., Gianoli M., Palmen M., Cerny S., Onan B., Singh S., Segers P., Bolcal C., Alhan C., Navarra E. (2022). Robotic coronary revascularization in Europe, state of art and future of EACTS-endorsed Robotic Cardiothoracic Surgery Taskforce. Interact. Cardiovasc. Thorac. Surg..

[41] Rufa M.I., Ursulescu A., Dippon J., Aktuerk D., Nagib R., Albert M., Franke U.F.W. (2024). Is minimally invasive multi-vessel off-pump coronary surgery as safe and effective as MIDCAB?. Front. Cardiovasc. Med..

[42] Zhou Z., Dilip K.A., Gleboff A., Nazem A., Green G.R., Cherney A., Lutz C.J. (2024). Comparison of Robot-Assisted Multivessel Minimally Invasive Direct Coronary Artery Bypass and Hybrid Revascularization. Ann. Thorac. Surg. Short. Rep..

[43] Hwang B., Ren J., Wang K., Williams M.L., Yan T.D. (2024). Systematic review and meta-analysis of two decades of reported outcomes for robotic coronary artery bypass grafting. Ann. Cardiothorac. Surg..

[44] Leonard J.R., Rahouma M., Abouarab A.A., Schwann A.N., Scuderi G., Lau C., Guy T.S., Demetres M., Puskas J.D., Taggart D.P. (2018). Totally endoscopic coronary artery bypass surgery: A meta-analysis of the current evidence. Int. J. Cardiol..

[45] Nisivaco S., Bhasin R., Kitahara H., Patel B., Coleman C., Grady K., Oh W.H., Balkhy H.H. (2024). Bilateral internal thoracic artery grafting in robotic beating-heart totally endoscopic coronary artery bypass: 10-year outcomes. Ann. Cardiothorac. Surg..

[46] Kofler M., Schachner T., Reinstadler S.J., Stastny L., Dumfarth J., Wiedemann D., Feuchtner G., Friedrich G., Bonatti J., Bonaros N. (2017). Comparative Analysis of Perioperative and Mid-Term Results of TECAB and MIDCAB for Revascularization of Anterior Wall. Innovations.

[47] Deppe A.C., Liakopoulos O.J., Choi Y.H., Slottosch I., Kuhn E.W., Scherner M., Stange S., Wahlers T. (2013). Endoscopic vein harvesting for coronary artery bypass grafting: A systematic review with meta-analysis of 27,789 patients. J. Surg. Res..

[48] Ferdinand F.D., MacDonald J.K., Balkhy H.H., Bisleri G., Hwang H.Y., Northrup P., Trimlett R.H.J., Wei L., Kiaii B.B. (2017). Endoscopic Conduit Harvest in Coronary Artery Bypass Grafting Surgery: An ISMICS Systematic Review and Consensus Conference Statements. Innovations.

[49] Williams J.B., Peterson E.D., Brennan J.M., Sedrakyan A., Tavris D., Alexander J.H., Lopes R.D., Dokholyan R.S., Zhao Y., O’Brien S.M. (2012). Association between endoscopic vs open vein-graft harvesting and mortality, wound complications, and cardiovascular events in patients undergoing CABG surgery. JAMA.

[50] Rosati F., Rattenni F., Boldini F., Bacco LDi Redaelli P., Benussi S. (2024). How I do it: Simplified Cox-Maze IV via right mini-thoracotomy. Ann. Cardiothorac. Surg..

[51] Muneretto C., Baudo M., Rosati F., Petruccelli R.D., Curnis A., Di Bacco L., Benussi S. (2023). Thoracoscopic Surgical Ablation of Lone Atrial Fibrillation: Long-term Outcomes at 7 Years. Ann. Thorac. Surg..

[52] Mack M.J., Leon M.B., Thourani V.H., Makkar R., Kodali S.K., Russo M., Kapadia S.R., Malaisrie S.C., Cohen D.J., Pibarot P. (2019). Transcatheter Aortic-Valve Replacement with a Balloon-Expandable Valve in Low-Risk Patients. N. Engl. J. Med..

[53] Hørsted Thyregod H.G., Jørgensen T.H., Ihlemann N., Steinbrüchel D.A., Nissen H., Kjeldsen B.J., Petursson P., De Backer O., Olsen P.S., Søndergaard L. (2024). Transcatheter or surgical aortic valve implantation: 10-year outcomes of the NOTION trial. Eur. Heart J..

[54] Blankenberg S., Seiffert M., Vonthein R., Baumgartner H., Bleiziffer S., Borger M.A., Choi Y.-H., Clemmensen P., Cremer J., Czerny M. (2024). Transcatheter or Surgical Treatment of Aortic-Valve Stenosis. N. Engl. J. Med..

[55] Mack M.J., Leon M.B., Thourani V.H., Pibarot P., Hahn R.T., Genereux P., Kodali S.K., Kapadia S.R., Cohen D.J., Pocock S.J. (2023). Transcatheter Aortic-Valve Replacement in Low-Risk Patients at Five Years. N. Engl. J. Med..

[56] Stone G.W., Lindenfeld J., Abraham W.T., Kar S., Lim D.S., Mishell J.M., Whisenant B., Grayburn P.A., Rinaldi M., Kapadia S.R. (2018). Transcatheter Mitral-Valve Repair in Patients with Heart Failure. N. Engl. J. Med..

[57] Muller D.W.M., Farivar R.S., Jansz P., Bae R., Walters D., Clarke A., Grayburn P.A., Stoler R.C., Dahle G., Rein K.A. (2017). Transcatheter Mitral Valve Replacement for Patients With Symptomatic Mitral Regurgitation: A Global Feasibility Trial. J. Am. Coll. Cardiol..

[58] Sorajja P., Moat N., Badhwar V., Walters D., Paone G., Bethea B., Bae R., Dahle G., Mumtaz M., Grayburn P. (2019). Initial Feasibility Study of a New Transcatheter Mitral Prosthesis: The First 100 Patients. J. Am. Coll. Cardiol..

[59] Webb J.G., Chuang A., Meier D., von Bardeleben R.S., Kodali S.K., Smith R.L., Hausleiter J., Ong G., Boone R., Ruf T. (2022). Transcatheter Tricuspid Valve Replacement With the EVOQUE System: 1-Year Outcomes of a Multicenter, First-in-Human Experience. JACC Cardiovasc. Interv..

[60] Hahn R.T., Makkar R., Thourani V.H., Makar M., Sharma R.P., Haeffele C., Davidson C.J., Narang A., O’Neill B., Lee J. (2025). Transcatheter Valve Replacement in Severe Tricuspid Regurgitation. N. Engl. J. Med..

[61] Arnold S.V., Hahn R.T., Thourani V.H., Makkar R., Makar M., Sharma R.P., Haeffele C., Davidson C.J., Narang A., O’Neill B. (2025). Quality of Life After Transcatheter Tricuspid Valve Replacement: 1-Year Results From TRISCEND II Pivotal Trial. J. Am. Coll. Cardiol..

[62] Lurz P., Stephan von Bardeleben R., Weber M., Sitges M., Sorajja P., Hausleiter J., Denti P., Trochu J.N., Nabauer M., Tang G.H.L. (2021). Transcatheter Edge-to-Edge Repair for Treatment of Tricuspid Regurgitation. J. Am. Coll. Cardiol..

[63] Taramasso M., Gavazzoni M., Maisano F. (2019). Is tricuspid regurgitation a prognostic interventional target or is it just an indicator of worst prognosis in heart failure patients?. Eur. Heart J..

[64] Muneretto C., Bisleri G., Rosati F., Krakor R., Giroletti L., Di Bacco L., Repossini A., Moltrasio M., Curnis A., Tondo C. (2017). European prospective multicentre study of hybrid thoracoscopic and transcatheter ablation of persistent atrial fibrillation: The HISTORIC-AF trial. Eur. J. Cardiothorac. Surg..

[65] Rosati F., Baudo M., D’Alonzo M., Bacco LDi Arabia G., Muneretto C. (2024). Hybrid strategies for stand-alone surgical ablation of atrial fibrillation. Ann. Cardiothorac. Surg..

[66] Nasso G., Vignaroli W., Dicandia C.D., Filannino P., Lembo G., Fiore F., Brigiani M.S., Greco E., Agrò F., Santarpino G. (2025). Hybrid Atrial Fibrillation Ablation: A Decade-Long Single-Center Experience. Rev. Cardiovasc. Med..

[67] Doll N., Weimar T., Kosior D.A., Bulava A., Mokracek A., Mönnig G., Sahu J., Hunter S., Wijffels M., Van Putte B. (2025). Durable effectiveness and safety of hybrid ablation versus catheter ablation: 2-year results from the randomized CEASE-AF trial. Eur. J. Cardiothorac. Surg..

[68] Rivera A., Braga M.A.P., Ternes C.M.P., Gewehr D.M., Villa Martignoni F., Dal Forno A., Locke A.H., d’Avila A. (2025). Hybrid ablation for persistent/long-standing persistent atrial fibrillation: A meta-analysis and trial sequential analysis of randomized controlled trials. J. Interv. Card. Electrophysiol..

[69] Hussain S., Grimster A.G., Woelk S.W., Gumm T.G., Yap J.Y., Lee R.L., Providencia R.P., Papageorgiou N.A., Ahsan S.A. (2025). The hybrid ablation approach to treating long-standing persistent atrial fibrillation: Real-world data from a tertiary institution. Eur. Heart J..

[70] DeLurgio D.B., Ferguson E., Gill J., Blauth C., Oza S., Mostovych M., Awasthi Y., Ndikintum N., Crossen K. (2020). Convergence of Epicardial and Endocardial RF Ablation for the Treatment of Symptomatic Persistent AF (CONVERGE Trial): Rationale and design. Am. Heart J..

[71] Wats K., Kiser A., Makati K., Sood N., DeLurgio D., Greenberg Y., Yang F. (2020). The Convergent Atrial Fibrillation Ablation Procedure: Evolution of a Multidisciplinary Approach to Atrial Fibrillation Management. Arrhythmia Electrophysiol. Rev..

[72] Delurgio D.B., Crossen K.J., Gill J., Blauth C., Oza S.R., Magnano A.R., Mostovych M.A., Halkos M.E., Tschopp D.R., Kerendi F. (2020). Hybrid Convergent Procedure for the Treatment of Persistent and Long-Standing Persistent Atrial Fibrillation: Results of CONVERGE Clinical Trial. Circ. Arrhythm. Electrophysiol..

[73] Mehr M., Taramasso M., Besler C., Ruf T., Connelly K.A., Weber M., Yzeiraj E., Schiavi D., Mangieri A., Vaskelyte L. (2019). 1-Year Outcomes After Edge-to-Edge Valve Repair for Symptomatic Tricuspid Regurgitation: Results From the TriValve Registry. JACC Cardiovasc. Interv..

[74] Burns D.J.P., Wierup P., Gillinov M. (2021). Minimally Invasive Mitral Surgery: Patient Selection and Technique. Cardiol. Clin..

[75] Ailawadi G., Agnihotri A.K., Mehall J.R., Alan Wolfe J., Hummel B.W., Fayers T.M., Saeid Farivar R., Grossi E.A., Sloane Guy T., Clark Hargrove W. (2016). Minimally Invasive Mitral Valve Surgery I: Patient Selection, Evaluation, and Planning. Innovations.

[76] Rosati F., Baudo M., Tomasi C., Scotti G., Pirola S., Mastroiacovo G., Polvani G., Bisleri G., Benussi S., Di Bacco L. (2025). Prediction Model for POstoperative atriaL fibrillAtion in caRdIac Surgery: The POLARIS Score. J. Clin. Med..

[77] Romiti S., Vinciguerra M., Saade W., Anso Cortajarena I., Greco E. (2020). Artificial Intelligence (AI) and Cardiovascular Diseases: An Unexpected Alliance. Cardiol. Res. Pract..

[78] Leivaditis V., Beltsios E., Papatriantafyllou A., Grapatsas K., Mulita F., Kontodimopoulos N., Baikoussis N.G., Tchabashvili L., Tasios K., Maroulis I. (2025). Artificial Intelligence in Cardiac Surgery: Transforming Outcomes and Shaping the Future. Clin. Pract..

[79] Sulague R.M., Beloy F.J., Medina J.R., Mortalla E.D., Cartojano T.D., Macapagal S., Kpodonu J. (2024). Artificial intelligence in cardiac surgery: A systematic review. World J. Surg..

[80] Serafini B., Kim L., Saour B.M., James R., Hannaford B., Hansen R., Kohno T., Monsky W., Seslar S.P. (2022). Exploring telerobotic cardiac catheter ablation in a rural community hospital: A pilot study. Cardiovasc. Digit. Health J..

[81] Xia S.B., Lu Q.S. (2021). Development status of telesurgery robotic system. Chin. J. Traumatol.-Engl. Ed..

[82] Reddy S.K., Saikali S., Gamal A., Moschovas M.C., Rogers T., Dohler M., Marescaux J., Patel V. (2025). Telesurgery: A Systematic Literature Review and Future Directions. Ann. Surg..

[83] Popma J.J., Deeb G.M., Yakubov S.J., Mumtaz M., Gada H., O’Hair D., Bajwa T., Heiser J.C., Merhi W., Kleiman N.S. (2019). Transcatheter Aortic-Valve Replacement with a Self-Expanding Valve in Low-Risk Patients. N. Engl. J. Med..

[84] Afilalo J., Alexander K.P., Mack M.J., Maurer M.S., Green P., Allen L.A., Popma J.J., Ferrucci L., Forman D.E. (2014). Frailty assessment in the cardiovascular care of older adults. J. Am. Coll. Cardiol..

[85] Vahanian A., Beyersdorf F., Praz F., Milojevic M., Baldus S., Bauersachs J., Capodanno D., Conradi L., De Bonis M., De Paulis R. (2022). 2021 ESC/EACTS Guidelines for the management of valvular heart disease: Developed by the Task Force for the management of valvular heart disease of the European Society of Cardiology (ESC) and the European Association for Cardio-Thoracic Surgery (EACTS). Rev. Esp. Cardiol. (Engl. Ed.).

[86] Kobayashi J., Baron S.J., Takagi K., Thompson C.A., Jiao X., Yamabe K. (2024). Cost-effectiveness analysis of transcatheter aortic valve implantation in aortic stenosis patients at low- and intermediate-surgical risk in Japan. J. Med. Econ..

[87] Chotnoppharatphatthara P., Yoodee V., Taesotikul S., Yadee J., Permsuwan U. (2023). Transcatheter aortic valve implantation in patients with severe symptomatic aortic valve stenosis: Systematic review of cost-effectiveness analysis. Eur. J. Health Econ..

[88] Otto C.M., Nishimura R.A., Bonow R.O., Carabello B.A., Erwin J.P., Gentile F., Jneid H., Krieger E.V., Mack M., McLeod C. (2021). 2020 ACC/AHA Guideline for the Management of Patients With Valvular Heart Disease: Executive Summary: A Report of the American College of Cardiology/American Heart Association Joint Committee on Clinical Practice Guidelines. Circulation.

